# Prevalence, risk factors, and potential symptoms of hyponatremia after spinal surgery in elderly patients

**DOI:** 10.1038/s41598-022-23583-1

**Published:** 2022-11-03

**Authors:** Yuki Kinoshita, Koji Tamai, Makoto Oka, Hasibullah Habibi, Hidetomi Terai, Masatoshi Hoshino, Hiromitsu Toyoda, Akinobu Suzuki, Shinji Takahashi, Hiroaki Nakamura

**Affiliations:** Department of Orthopaedic Surgery, Osaka Metropolitan University Graduate School of Medicine, 1-5-7, Asahimachi, Abenoku, Osaka, Osaka 545-8585 Japan

**Keywords:** Geriatrics, Neurology, Neurological disorders

## Abstract

Old age and spinal surgery significantly increase the risk of postoperative hyponatremia. However, detailed analyses of postoperative hyponatremia after spinal surgery in elderly patients are lacking. Therefore, we retrospectively reviewed the records of 582 consecutive patients aged > 60 years who underwent spinal surgery to evaluate the frequency, risk factors, and symptoms of postoperative hyponatremia after spinal surgery in the elderly population. Postoperative hyponatremia was defined as a postoperative blood sodium level < 135 meq/L at postoperative day (POD)1, POD3, and/or after POD6. A total of 92 (15.8%) patients showed postoperative hyponatremia. On a multivariate analysis, a diagnosis of a spinal tumor/infection, decompression and fusion surgery, and lower preoperative sodium levels were significant independent factors of postoperative hyponatremia (p = 0.014, 0.009, and < 0.001, respectively). In total, 47/92 (51%) cases could have been symptomatic; vomiting was noted in 34 cases (37%), nausea in 19 cases (21%), headache in 14 cases (15%), and disturbances in consciousness, including delirium, in ten cases (21%); all incidences of these symptoms were significantly higher in elderly patients with postoperative hyponatremia than in the matched control group without postoperative hyponatremia (p < 0.05, respectively). Additionally, the length of stay was 2 days longer in patients than in the matched controls (p = 0.002).

## Introduction

The global average life expectancy increased by 5.5 years from 2000 to 2016^1^. Reflecting this trend, patients treated with surgical therapy for spinal diseases are becoming older in many countries. In fact, a report from Japan, which has the greatest aging population worldwide, demonstrated that the proportion of patients aged > 70 years among all spinal surgical cases increased from 25% in 2004 to 40% in 2015 and that the mean age at surgery increased from 54.6 years in 2004 to 63.7 years in 2015^2^.

Although progress in surgical techniques and equipment, as well as perioperative clinical care, have rendered surgery safer, it is still necessary to pay attention to postoperative complications, especially in elderly patients. One of the most common, however silently, complication is postoperative hyponatremia. Mild-to-severe hyponatremia may present with symptoms such as nausea, headache, gait instability, attention impairment, and falls, which may be considered as normal postoperative reactions^3–5^. Moreover, even mild hyponatremia in hospitalized patients is significantly correlated with 1-year and 5-year mortality^6^. A previous study reported that for every 1-mEq/L decrease in the sodium level below 135 mEq/L, mortality increases by 23%^7^. Furthermore, hyponatremia is correlated with perioperative complications, including pneumonia, wound infections, and major coronary events, as well as prolonged hospitalization^8^.

The prevalence of hyponatremia after general orthopedic surgery is as high as 30%^9^. Although several factors, including diuretic use, hypotonic fluids, and nonsteroidal anti-inflammatory drugs, have been reported as risk factors of postoperative hyponatremia^10–14^, the major factors that significantly increase the risk of postoperative hyponatremia are older age and spinal surgery^9^. However, no study has examined the frequency, risk factors, and potential symptoms of postoperative hyponatremia in elderly patients undergoing spinal surgery. Thus, this study aimed to (1) determine the frequency and risk factors for postoperative hyponatremia in this population and (2) evaluate the clinical symptoms and outcomes of postoperative hyponatremia by comparing patients with and without it.

## Methods

### Ethical statements

This study comprised a retrospective analysis of prospectively collected data on patients who underwent spine surgery in our institution. All study participants provided informed consent, and the study protocol was approved by the Institutional Review Board of Osaka City University (approval number: 3170). All methods were performed in accordance with the Declaration of Helsinki and the Ethical Guidelines for Medical and Health Research Involving Human Subjects in Japan. No funds were received in support of this work. No benefits in any form have been or will be received from a commercial party related directly or indirectly to the subject of this manuscript.

### Patient population

We retrospectively reviewed the medical records of 600 consecutive patients who underwent spinal surgery under general anesthesia at our institution between January 2018 and July 2020, were aged > 60 years by the time of operation, and had complete blood test data preoperatively, at postoperative day (POD)1, POD3, and POD6. Among these patients, those who underwent hemodialysis for chronic renal failure or underwent spinal surgery twice within 1 month, regardless of whether it was expected or unexpected, were excluded from analysis. Finally, a total of 582 patients were included in the current study (279 women and 303 men; mean age at surgery, 73.5 ± 7.4 years).

### Surgical indication

The surgical indication and approach are decided on a case-by-case basis by the treating physicians. Generally, patients with relatively slow progressive diseases such as lumbar spinal stenosis or cervical spondylotic myelopathy are initially treated with conservative therapy. Surgery is performed if patients do not achieve satisfactory improvement. Patients with radiological instability are treated with fusion surgery^15,16^, and those with emergent symptoms such as severe palsy, severe myelopathy, infection, and progressive tumors are treated with adequate surgery immediately. In all cases in this study, seven surgeons were involved in the decision making regarding surgical indication and approach in the experimental period; however, the final surgical indication and approach were decided through a consensus of all surgeons in our institution.

### Perioperative clinical care

#### Postoperative intravenous infusions

As part of our clinical pathway, we administered 1000 mL of acetated Ringer's solution and 1000 mL of glucose-acetated maintenance solution postoperatively from the day of surgery to the next day with some adjustments based on each patient’s weight, renal function, and blood loss. When a patient's food intake exceeded 50%, intravenous treatment was considered as complete on the next morning.

#### Postoperative painkillers

The standard care protocol included the routine use of celecoxib 200 mg/day as a painkiller for 7 days postoperatively. The use of additional painkillers, such as acetaminophen, opioids, or non-steroidal anti-inflammatory drugs (oral or intravenous administration), was allowed as needed. In patients with chronic renal dysfunction, the pharmacological dose was adjusted based on the creatinine clearance value.

### Collected data

#### Preoperative data

Data regarding the age at surgery; sex; height; weight; body mass index (BMI); comorbidities (diabetes mellitus, rheumatoid arthritis, and osteoporosis); use of medications that could affect blood sodium levels, including steroids, anticoagulants, and angiotensin II receptor blockers (ARBs)^17^; and type of spinal disease, that is, degenerative lumbar disease, degenerative thoracic disease, degenerative cervical disease, degenerative scoliosis, spinal tumor/infection; and so on (including implant removal) were collected.

#### Operative data

Operative data regarding the total operative time, blood loss (mL), surgical method (decompression or decompression and fusion), and surgical complications (dural tear) were collected.

#### Postoperative data

Postoperative data regarding the potential symptoms of postoperative hyponatremia, including vomiting, nausea, leg edema, headache, and a disturbance of consciousness, were evaluated retrospectively from medical records. Data on the length of hospital stay, discharge to home rate, and incidence of systemic complications, including circulatory disease and respiratory disease, were collected.

#### Blood test data

Blood test data regarding the estimated glomerular filtration rate (eGFR) and levels of hemoglobin (Hb) (g/dL), hematocrit (Hct) (%), platelet (Plt) (× 10,000/μ), albumin (Alb) (g/dL), urea nitrogen (UN) (mg/dL), creatinine (Cre) (mg/dL), sodium (Na) (mEq/L), potassium (K) (mEq/L), and hemoglobin A1c (HbA1c) (%) were collected. Blood tests were performed preoperatively, and on POD1, POD3, and POD6 in all patients. Blood tests performed after POD6 were evaluated as “after POD6”.

### Definition of hyponatremia

Postoperative hyponatremia, according to clinical guidelines, was defined as a postoperative blood sodium level < 135 meq/L at POD1, POD3, or after POD6^18,19^. When patients showed hyponatremia < 135 meq/L preoperatively but not postoperatively, the patient was considered to show non-postoperative hyponatremia. Additionally, when patients showed hyponatremia < 135 meq/L both preoperatively and postoperatively, regardless of the POD, the patient was considered to show postoperative hyponatremia.

### Study design & statistical analysis

The incidence of postoperative hyponatremia was determined. Subsequently, three analyses were performed.

1. Risk factor analysis
  To calculate the risk factors of postoperative hyponatremia, all patients were divided into two groups; the postoperatively hyponatremia and control groups. Preoperative, operative, and postoperative factors, as well as preoperative blood test data, were compared between groups using the Mann–Whitney U test for continuous variables and the chi-squared test for categorical variables. Subsequently, variables with a significance of p < 0.05 on the univariate analysis were included in a multivariate analysis as explanatory variables; postoperative hyponatremia was set as the objective variable. The adjusted odds ratio (aOR) and 95% confidence intervals (CIs) of dependent variables were calculated.
2. Potential symptoms and outcomes analysis
  To adjust the differences of backgrounds between the postoperative hyponatremia and control groups, the matched control group was created using propensity score matching. To estimate the propensity score, a logistic regression model was fitted using the patient’s age, sex, type of spinal disease, and surgical method data. The nearest-neighbor matching procedure was used, with the restriction that the matched propensities had to be within 0.01 units of each other. To identify the characteristics associated with the postoperative hyponatremia group, the clinical symptoms and clinical outcomes of patients in this group were compared with participants in the matched control group using the chi-squared test.
3. Characteristics of the symptomatic hyponatremia analysis
  TO identify the factors related to symptomatic hyponatremia, the postoperative hyponatremia group was further classified into two subgroups; the symptomatic group, which included hyponatremia patients with potential symptoms, and the asymptomatic group, which included hyponatremia patients without any potential symptoms. Preoperative data, operative data, and blood test data were compared between the symptomatic and asymptomatic groups using the Mann–Whitney U test for continuous variables and the chi-squared test for categorical variables.
  Continuous variables were represented by the average ± 1.0 standard deviation when showing normal distribution; otherwise, they were represented as medians with the 1st and 3rd quartiles. All analyses were performed using SPSS version 23 (SPSS, Chicago, IL, USA). A p-value of < 0.05 was considered statistically significant.

## Results

### Incidence of postoperative hyponatremia

**Figure 1:**
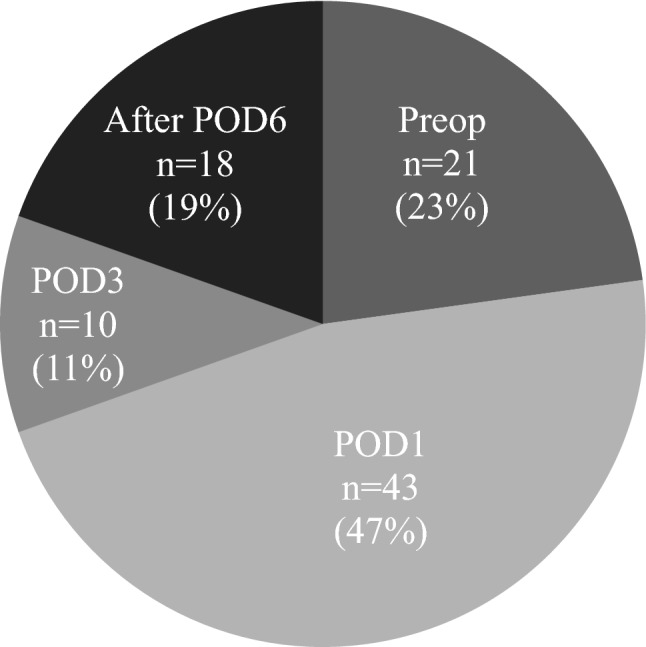
The timing of postoperative hyponatremia is shown.

Of the 582 patients in this analysis, 92 patients (15.8%) showed postoperative hyponatremia, regardless of onset timing. Among these, 21 patients showed hyponatremia both preoperatively and postoperatively, 43 patients showed hyponatremia from POD1, 10 patients showed hyponatremia from POD3, and 18 patients showed hyponatremia after POD6 (Fig. 1).

### Risk factors of postoperative hyponatremia

There were no significant differences between both groups in preoperative factors, with the exception of anticoagulant use (Table1); patients with preoperative hyponatremia took anticoagulants more frequently than control participants (p < 0.001). Regarding surgical characteristics, the method and spinal disease were significantly different between both groups (p = 0.002 and 0.010, respectively; Table 2). Regarding preoperative laboratory data, there were significant differences between groups in some preoperative blood test parameters, including Hct, Alb, and Na (p = 0.002, 0.013, and < 0.001, respectively; Table 3). The multivariate analysis, which included significant variables taken from the univariate analysis, revealed a diagnosis of spinal tumor/infection, decompression and fusion surgery, and preoperative lower Na level as significant independent factors (p = 0.014, 0.009, and < 0.001, respectively; Table 4). Namely, patients who underwent spinal surgery for tumor/infection had a significantly higher incidence of postoperative hyponatremia than those who underwent spinal surgery for lumbar disease (aOR, 3.15; p = 0.014). Patients who underwent decompression and fusion surgery had a significantly higher incidence of postoperative hyponatremia than those who underwent decompression surgery (aOR, 2.15; p = 0.009). Patients with a lower preoperative sodium level had a significantly higher incidence of postoperative hyponatremia than patients with higher preoperative sodium levels (aOR, 0.64; p < 0.001).

**Table 1:** Patient characteristics.

	Control group	Postop hyponatremia group	p-value
Numbers	490	92	
Average years	74 (63, 80)	75.5 (69, 78)	0.400*
Sex (female/male)	238/252	41/51	0.482^#^
Height (cm)	156.5 ± 10.5	155.8 ± 10.1	0.504*
Weight (kg)	58.6 ± 12.2	56.9 ± 12.1	0.212*
BMI (kg/m^2^)	23.8 ± 3.7	23.4 ± 4.2	0.384*
**Comorbidity**			
Diabetes	52	13	0.370^#^
Rheumatoid arthritis	26	5	0.960^#^
Osteoporosis	52	10	0.942^#^
**Preop medicine**			
Steroids	25	5	0.897^#^
Anticoagulant	120	41	< 0.001^#^
ARB	173	36	0.509^#^

Continuous variables, except for age, were represented by and average ± 1.0 standard deviation.

*ARB* angiotensin II receptor blocker, *BMI* body mass index, *Preop* preoperative, *Postop* postoperative.

*Mann–Whitney U test.

^#^Chi-squared test.

**Table 2:** Surgical characteristics.

	Control group (n = 490)	Postop hyponatremia group (n = 92)	p-value
**Type of spinal disease**			0.010^#^
Cervical	109	20	
Thoracic	8	2	
Lumbar	315	50	
Scoliosis	22	5	
Tumor/infection	25	14	
**Surgical method**			0.002^#^
Decomp	331	46	
Fusion + decomp	159	46	
Surgical time (min)	146 (105, 205)	153 (114, 203)	0.830*
Blood loss (mL)	70 (20, 175)	86 (30, 208)	0.396*
**Complications**			
Dural tear	14	4	0.652^#^

Continuous variables were represented by the median with 1st and 3rd quartiles.

*Decomp* decompression, *Postop* postoperative.

*Mann–Whitney U test.

^#^Chi-squared test.

**Table 3:** Preoperative laboratory data.

	Control group (n = 490)	Postop hyponatremia group (n = 92)	p-value
Hb (g/dL)	13.3 ± 1.6	12.9 ± 1.8	0.066
Hct (%)	39.2 ± 4.9	37.5 ± 4.9	0.002
Plt (× 10,000/μ)	23.0 ± 15.0	23.7 ± 8.4	0.401
Alb (g/dL)	4.0 ± 0.4	3.8 ± 0.6	0.013
UN (mg/dL)	18.6 ± 7.8	18.1 ± 8.8	0.670
Cr (mg/dL)	0.87 ± 0.53	0.84 ± 0.40	0.461
eGFR	65.1 ± 19.0	69.7 ± 24.0	0.091
Na (mEq/L)	141.0 ± 1.9	137.8 ± 4.0	< 0.001
K (mEq/L)	4.3 ± 0.41	4.3 ± 0.47	0.557
HbA1c (%)	6.0 ± 0.68	6.0 ± 0.67	0.876

p-values were determined by the Mann–Whitney U test. Variables were represented by average ± 1.0 standard deviation.

*Alb* albumin, *Cre* creatinine, *eGFR* estimated glomerular filtration rate, *Hb* hemoglobin, *HbA1c* hemoglobin A1c, *Hct* hematocrit, *K* potassium, *Na* sodium, *Plt* platelet, *Postop* postoperative, *UN* urea nitrogen.

**Table 4:** Multivariate analysis for the risk factors of postoperative hyponatremia.

Explanatory variables		Reference	aOR	P-value	95% CI
Preop medicine	Anticoagulant	Non	1.64	0.094	0.92–2.92
Type of spinal disease	Cervical	Lumbar	1.00	0.988	0.52–1.92
	Thoracic	Lumbar	1.86	0.482	0.33–10.6
	Scoliosis	Lumbar	0.94	0.915	0.30–2.97
	Tumor/infection	Lumbar	3.15	< 0.05	1.26–7.84
Surgical method	Fusion + decomp	Decomp	2.15	< 0.05	1.21–3.84
Preop lab data	Hct	Continuous	0.95	0.106	0.89–1.01
	Alb	Continuous	1.09	0.782	0.59–2.03
	Na	Continuous	0.64	< 0.001	0.57–0.72

*Alb* albumin, *aOR* adjusted odds ratio, *CI* confidence interval, *Decomp* decompression, *Hct* hematocrit, *Na* sodium, *Preop* preoperative.

### Potential symptoms and outcomes of postoperative hyponatremia

After matching age, sex, type of spinal disease, and surgical method, 92 patients were extracted to the matched control group from the control group using propensity score analysis. There were no significant differences between the postoperative hyponatremia group and matched control group in age (p = 0.340), sex (p = 0.659), type of spinal disease (p = 0.819), and surgical method (p = 0.769). In the postoperative hyponatremia group, 34 patients demonstrated postoperative vomiting (37%), 19 patients demonstrated nausea (21%), 14 patients demonstrated headache (15%), 6 patients demonstrated leg edema (7%), and 10 patients had disturbance of consciousness including delirium (11%). All such incidences of potential symptoms, except for leg edema, were significantly higher in the postoperative hyponatremia group than the matched control group (Table 5). Regarding clinical outcomes, no significant differences in discharge to home rates and perioperative systemic complications were observed between both groups. However, the postoperative hyponatremia group demonstrated a 2-day longer hospital stay than the matched control group (p = 0.022).

**Table 5:** Potential symptoms and outcomes of hyponatremia postoperatively.

	Matched control group (n = 92)	Postop hyponatremia group (n = 92)	p-value
**Symptoms**			
Vomiting	7	19	0.018^#^
Nausea	14	34	0.001^#^
Leg edema	4	6	0.747^#^
Headache	1	14	0.001^#^
Consciousness disturbance	1	10	0.009^#^
Any symptoms	19	47	< 0.001^#^
**Outcomes**			
Length of stay (days)	15 (11, 20)	17 (12, 21)	0.022*
Discharge to home (%)	71.9	65.2	0.420^#^
Systemic complication			0.504^#^
Cardiac disease	1	0	
Respiratory disease	2	3	
Others	4	6	

Continuous variables are represented by their median with 1st and 3rd quartiles.

*Postop* postoperatively.

*Mann–Whitney U test.

^#^Chi-squared test.

### Factors relating with symptomatic hyponatremia

Forty-seven patients were included in the symptomatic group, and 45 patients were included in the asymptomatic group. In the comparison between the symptomatic and asymptomatic groups, no significant differences were noted (Table 6).

**Table 6:** Sub-analysis of the factors relating to symptomatic hyponatremia.

	Asymptomatic group	Symptomatic group	P-value
Numbers	45	47	
Average years	76 (68.5, 79.5)	75 (68, 79)	0.902*
Sex (female/male)	28/17	23/24	0.216^#^
BMI (kg/m^2^)	23.1 ± 4.2	23.7 ± 4.1	0.515*
Diabetes	8	5	0.380^#^
**Type of spinal disease**			0.242^#^
Cervical	13	7	
Thoracic	0	2	
Lumbar	21	29	
Scoliosis	2	3	
Tumor/infection	8	6	
**Surgical method**			0.677^#^
Decomp	24	22	
Fusion + decomp	21	25	
**Preoperative blood data**			
Hb (g/dL)	13.1 ± 1.7	12.7 ± 1.9	0.302*
Hct (%)	38.2 ± 4.4	36.9 ± 5.1	0.170*
Plt (× 10,000/μ)	25.0 ± 7.3	23.0 ± 9.4	0.264*
Alb (g/dL)	3.9 ± 0.7	3.8 ± 0.6	0.770*
UN (mg/dL)	17.7 ± 8.0	18.6 ± 9.5	0.633*
Cr (mg/dL)	0.86 ± 0.41	0.81 ± 0.39	0.588*
eGFR	70.2 ± 25.7	69.2 ± 22.4	0.846*
Na (mEq/L)	137.0 ± 4.1	138.5 ± 3.9	0.088*
K (mEq/L)	4.4 ± 0.4	4.2 ± 0.5	0.241*
HbA1c (%)	6.1 ± 0.7	5.9 ± 0.6	0.360*

Continuous variables were represented by their average ± 1.0 standard deviation when showing normal distribution; otherwise, they were represented by medians with 1st and 3rd quartiles.

*BMI* body mass index, *Decomp* decompression, *Alb* albumin, *Cre* creatinine, *eGFR* estimated glomerular filtration rate, *Hb* hemoglobin, *HbA1c* hemoglobin A1c, *Hct* hematocrit, *K* potassium, *Na* sodium, *Plt* platelet, *Postop* postoperative, *UN* urea nitrogen.

*Mann–Whitney U test.

^#^Chi-squared test.

### An illustrative case

The patient was a 77-year-old woman who underwent L4-5 lateral interbody fusion for lumbar spinal canal stenosis with instability. The patient showed mild hyponatremia (129 mEq/L) on POD3. Hence, she was treated with oral salt loading (3 g/day) until POD7 when her blood sodium level improved to 131 mEq/L. However, disturbance of consciousness suddenly appeared after rehabilitation at POD12 (E2V1M4 of Glasgow Coma Scale). Severe hyponatremia (116 mEq/L) and hyperkalemia (5.3 mEq/L) in the blood test, but normal natrium (70 mmol/L) and kalium (37 mmol/l) concentration in a urine test, were found. There were no abnormal findings on radiography examinations such as brain magnetic resonance imaging. Endocrinologists diagnosed this patient with mineralocorticoid responsive hyponatremia of the elderly (MRHE), which is a condition in which elderly patients taking ARBs and non-steroidal anti-inflammatory drugs (NSAIDs) develop severe hyponatremia following dehydration.^20^ This patient routinely took NSAIDs and ARBs and seemed to be dehydrated after rehabilitation. Her medications were discontinued, and we administered 1.7% saline at a dose of 45 mL/h. The next day, her disturbance of consciousness improved (E4V5M6), and blood sodium concentration was elevated to be 124 mEq/L. Treatment using 1.2% saline administration, oral salt loading (3 g/day), and prescribed fludrocortisone was continued. Finally, the blood sodium level reached 133 mEq/L on POD22, and the patient was discharged.

## Discussion

In the current study, we found that the incidence of hyponatremia after spinal surgery in elderly patients was 15.8%. The risk factors for postoperative hyponatremia included a specific disease type (tumor/infection) and surgical method (decompression and fusion) and low preoperative sodium levels. Furthermore, more than 50% of elderly patients with postoperative hyponatremia could be symptomatic, and symptoms include disturbances in consciousness. The length of hospital stay was 2 days longer in patients with hyponatremia than in matched control patients.

The frequency of postoperative hyponatremia in the field of orthopedics in previous reports was approximately 30%.^9^ However, in the current study, which selected patients with two major high-risk factors of preoperative hyponatremia, the incidence was 15.8%. This discrepancy could be due to advances in anesthesia and perioperative management, including infusion management, clinical pathways, and medication selection. Regarding the onset of postoperative hyponatremia, a previous report demonstrated that 93% of cases were found within 48 h^9^. In contrast, in the current study, approximately 20% of preoperative hyponatremia cases were observed after POD6. The postulated reason for this difference is that the previous report was from the United States, whereas the postoperative hospital stay was shorter than that in the current study. Therefore, the observation period may have been shortened, consequently increasing the ratio of cases with early-onset hyponatremia.

Old age, spinal surgery, and hip surgery have been reported as risk factors for postoperative hyponatremia^20^. In the current study, which focused on elderly patients who underwent spinal surgery, the risk factors for postoperative hyponatremia were a diagnosis of spinal tumor/infection, decompression and fusion surgery, and low preoperative sodium level. The incidence of postoperative complications is known to be higher in decompression and fusion surgery than in decompression surgery, partially because of the higher invasiveness of fusion surgery^21–24^. Furthermore, the incidence of hyponatremia is higher with more invasive surgeries^9,25–27^. These data suggest that hyponatremia is more likely to occur in decompression and fusion surgery than in decompression alone due to higher surgical invasiveness. Regarding disease type, a previous report revealed that 21% of patients with a severe infection and 14% of patients with a malignant tumor showed hyponatremia^27^. This suggests that a spinal tumor or spinal infection sets the potential for hyponatremia in the patient background. Additionally, adding surgical invasion to background details may make hyponatremia apparent via MRHE or syndrome of inappropriate secretion of antidiuretic hormone^3^.

We retrospectively evaluated vomiting, nausea, headache, leg edema and disturbances in consciousness, including delirium, which were reported as potential symptoms of postoperative hyponatremia^21^. We found that the incidence of such potential symptoms except for leg edema were significantly higher in patients with hyponatremia than in the matched controls. In a prospective multicenter study, the incidence of observed symptoms of hyponatremia were reported as follows: vomiting, 30%; nausea, 44%; headache, 27%; general fatigue, 59%; and disturbances in consciousness, 5%^28^. The incidences of symptoms of postoperative hyponatremia in the current study (vomiting, 37%; nausea, 21%; headache, 15%; and disturbances in consciousness, 11%) were similar to those in a previous report. Additionally, we found that the length of hospital stay was 2 days longer in patients with hyponatremia than in the matched controls. Although the retrospective nature of this study rendered it difficult to exclude information bias, the finding that 50% of patients with hyponatremia were likely to be symptomatic may comprise an important message for spine physicians.

The clinical implications relevant to spine surgeons of this study are as follows: hyponatremia in elderly patients who undergo spinal surgery is not uncommon and can have adverse outcomes, and approximately 50% of patients with postoperative hyponatremia are likely to be symptomatic. Moreover, we have revealed some risk factors of postoperative hyponatremia in elderly patients. Due to differences in healthcare systems among countries, spinal surgeons might not be able to follow-up with patients for a long time postoperatively. However, by sharing the current findings with home doctors and attending surgeons, patients who show postoperative hyponatremia may more likely be treated with adequate early and appropriate treatment.

Several limitations of this study should be addressed. First, as described above, this was a retrospective study. Hence, the postoperative interventions were not identical among patients, data on perioperative infusions were not analyzed, and there might be some unreliability in symptom data. Secondly, we could not evaluate the causes of postoperative hyponatremia or adequateness of interventions after the onset of hyponatremia. Third, we analyzed data from only one institution. Therefore, a prospective multicenter study to evaluate the incidence, cause, and adequate therapy for postoperative hyponatremia in the elderly population is necessary.

## Conclusion

The incidence of hyponatremia after spinal surgery in the elderly population is nearly 16%, and risk factors include a diagnosis of a spinal tumor/infection, fusion surgery, and low preoperative sodium levels. Additionally, more than 50% of patients with postoperative hyponatremia could be symptomatic, and such patients need a 2-day longer length of hospital stay than matched controls. By sharing the current findings with home doctors and attending surgeons, patients who show postoperative hyponatremia can more likely receive appropriate treatment with adequate timing.

## Data Availability

The datasets used and/or analyzed during the current study are available from the corresponding author on reasonable request.
